# Biomarkers in Ovarian Pathology: From Screening to Diagnosis. Review of the Literature

**DOI:** 10.3390/jpm11111115

**Published:** 2021-10-29

**Authors:** Miguel Ángel Elorriaga, José Luis Neyro, Jon Mieza, Ignacio Cristóbal, Antoni Llueca

**Affiliations:** 1 Servicio de Ginecología y Obstetricia, Hospital Universitario Cruces, Universidad del País Vasco, EHU—UPV, 48903 Baracaldo, Spain; melorriagag@gmail.com (M.Á.E.); doctorneyro@gmail.com (J.L.N.); jonmieza@yahoo.es (J.M.); 2 Internacional de Climaterio y Menopausia, Universidad a Distancia de Madrid (UDIMA) y Universidad Veracruzana Lomas del Estadio S/N, Col. Zona Universitaria C.P. 91090, Xalapa, Mexico; 3 Instituto Ginecológico Deusto, 48014 Bilbao, Spain; 4 Servicio de Obstetricia y Ginecología, Hospital Clínico San Carlos, Universidad Francisco de Vitoria, 28223 Madrid, Spain; icristobalg@sego.es; 5 Unidad de Referencia en Cirugía Oncológica Abdomino-Pélvica (UR-COAP), Hospital General Universitario de Castellón, 12004 Castelló, Spain; 6 Departamento de Medicina, University Jaume I (UJI), 12071 Castellón, Spain; 7 University Jaume I (UJI), Av de Vicent Sos Baynat s/n, 12071 Castellón, Spain

**Keywords:** biomarkers, tumour markers, CA125, HE4 antigen, algorithms, screening, ovarian cancer

## Abstract

Background: Ovarian cancer has a low incidence, but high mortality due to a habitual diagnosis in advanced cancer stages. Currently, used biomarkers have good sensitivity, but low specificity. Aim: To determine the usefulness of the biomarkers and algorithms used up to now in the screening, diagnosis, response to treatments and identification of recurrence in patients with ovarian masses. Methodology: Systematic search of publications in English in the Medline-PubMed database with the terms: “biomarkers”, “tumour”, “tumour biomarkers”, “marker”, “tumour marker”, “ovarian cancer”, “ovarian”, “Neoplasms”, “cancer”, CA-125 Antigen; Human Epididymis-specific Protein E4; Risk of Malignancy Index (RMI); Risk of Ovarian Malignancy Algorithm (ROMA); Ovarian Neoplasms. Original articles, clinical trials, reviews, systematic reviews and meta-analyses, published between January 2000 and November 2020, were selected to determine the usefulness (among others) of CA 125 and HE4 antigen in ovarian cancer. Results: Finally, 39 transcendental publications were selected to write this article to determine the usefulness of tumour markers and algorithms in ovarian cancer. Conclusions: The usefulness of the tumour markers antigen CA125 and antigen HE4 individually or as a basis for decision-making algorithms has low specificity; however, there is little evidence that confirms their usefulness as markers in ovarian cancer screening.

## 1. Introduction

There have been promising advances in ovarian cancer (OC) research. However, to our understanding, there is still a lot to discover about the various subtypes of cancer, the different risk factors, and genetic mutations or the diverse biological behaviours and different prognoses.

The origin of the various types of OC is not definitively clarified, we do not know how they develop, and these circumstances impede clinical progress in the prevention, early detection, treatment, and management of OC. In recent decades, a marginal reduction in morbidity and mortality has been achieved because of a series of factors, such as the pathology’s global investigation. However, without attending to the different histological subtypes, which has resulted in the different screening platforms have not proven to be applied effectively, lacking validation.

In other tumour pathologies, symptomatology facilitates and guides the diagnosis, which is not the case in OC. Unlike other gynaecological cancers, where early symptoms lead to an early diagnosis, in OC, the symptoms do not appear until advanced stages. This is an essential factor contributing to the high mortality rate, especially in women with high-grade serous carcinoma (HGSC), the most common and lethal subtype. Approximately two-thirds of women with OC are diagnosed with advanced-stage or definitively unstageable cancer, and the 5-year survival rate for these cases is less than 30% [1].

The most common type of diagnosed cancer in women in Spain during 2020 have been breast, colon and rectum cancers. These are followed by lung, uterine corpus uteri, urinary bladder, non-Hodgkin’s lymphomas and thyroid cancer, all of which account for more than 4000 cases per year. In women, the highest number of tumoral deaths in Spain were breast (up 0.7%), colon and rectum (down 3.8%) and lung. Although OC is relatively rare, it is one of the most lethal, accounting for almost 12 new cases per 100,000 women and 2.6% of all new cancer cases in women. In Spain, 3645 women will be diagnosed with OC in 2020, of whom 1949 will die, representing 53.47% of affected women. In absolute terms, this is 7.7 deaths per 100,000 women and represents 5.1% of all cancer deaths in Spain. In terms of survival rate, it is 45.6% at 5 years (90% in breast cancer). The high mortality and low survival rates of OC can be attributed to the fact that it is rarely diagnosed at an early stage and hence the importance of the search for sensitive biomarkers in these early stages. In fact, in 60% of cases, the advanced disease is diagnosed when the neoplasm has spread beyond the ovary to distant organs or lymph nodes [2].

Only a small fraction of OC cases appears to originate in the ovary. A paradigm shift is occurring, modifying the focus away from viewing the ovaries as the origin in most cases [3]. This has important clinical implications. On this premise, performing prophylactic salpingectomy may prove to be a strategy to reduce the incidence (and mortality) of OC, and knowing where these early precursor lesions start may help to guide the development of new imaging and other techniques to detect precursor lesions before they spread to the ovaries and elsewhere [4].

The term ovarian cancer technically represents various subtypes to emphasise the heterogeneity of the disease and all its histopathological subtypes. The main subtypes of OC are named according to the cells of the epithelia from which they derive, thus we will find serous, endometrioid, mucinous and clear cell carcinomas, depending on how they resemble the epithelia of the tubes, endometrium, endocervix or the gestational endometrium, respectively [5].

This article provides an overview of the current state of ovarian cancer biomarker research, highlighting the main gaps in this knowledge and providing recommendations that could help reduce OC morbidity and mortality by focusing on promising research topics and technologies that could promote early detection.

Therefore, this paper aims to review the means available to make the earliest possible diagnosis, improving survival and quality of life. The study of epithelial OC biomarkers is thus specifically targeted because this subtype accounts for 90% of all OCs and is responsible for most OC-related deaths.

## 2. Methodology

**Figure 1:**
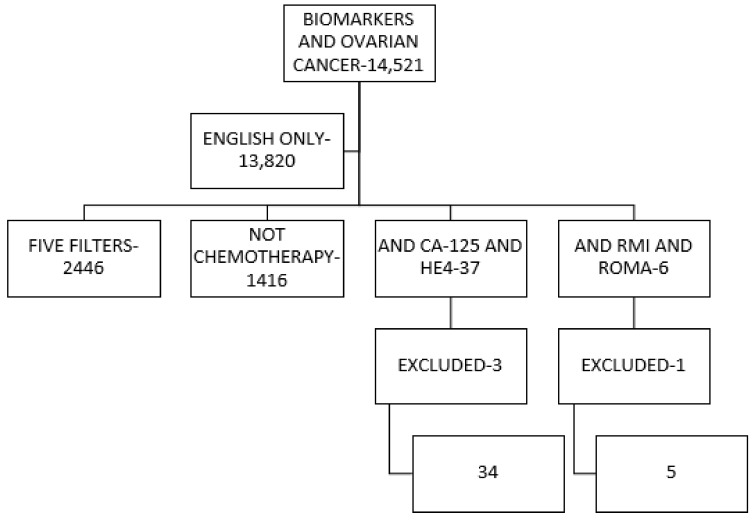
Summary of the literature search shown in the flow chart. A search was performed using the keywords “BIOMARKERS AND OVARIAN CANCER” to narrow the options. After excluding all studies published in languages other than English (13,820), five filters were applied to the search: 1. Clinical Trials, 2. Meta-Analysis, 3. Randomised Controlled Trials, 4. Reviews, and 5. Systematic Reviews. All studies referring to OC chemotherapy (1416) were also excluded, as they were not part of our objective. In the CA125 and HE4 group, studies were excluded because they were follow-up studies (1), or because they focused only on high-risk women (1), or because they were comparative between mesothelioma and HE4 (1), all of them different from the objectives of this work. ROMA: risk of ovarian malignancy algorithm. RMI: Risk of Malignancy Index for Ovarian Cancer. For the same reason, in the RMI and ROMA group, another study was excluded because it was an ultrasound-only analysis. Finally, 39 studies were obtained for complete analysis.

A search of publications in the Medline-PubMed database was carried out by selecting the following terms using MeSH (Medical Subject Headings): “biomarkers”, “tumour”, “tumour biomarkers”, “marker”, “tumour marker”, “ovarian cancer”, “ovarian”, “neoplasms”, “cancer”, “CA125”, “HE4”, “algorithms”. The search was limited to English, selecting only English articles, clinical trials, reviews, systematic reviews and meta-analyses, published between January 2000 and November 2020 that determined the usefulness of CA125 and HE4 antigen in ovarian cancers. A similar search was performed in the Web of Science databases. Figure 1 shows [6] the literature search filters and summarises the selection made.

A biomarker is a biological molecule present in blood, body fluids or tissues that signs a standard or abnormal process, condition, or disease. A biomarker can be used to monitor the body’s response to a treatment for a disease [7]. Although the most commonly used biomarkers in clinical care are proteins, the definition includes a broad spectrum of biochemical substances, including nucleic acids (e.g., DNA and various types of RNA), lipids, small metabolites and even full cells [8].

Predictive biomarkers are used in risk assessment and to measure biological responses to intervention; prognostic biomarkers are used to describe outcomes such as overall or progression-free survival.

## 3. Results

### 3.1. Tumour Marker CA125 

CA125 gained a reputation after a study identified an antibody to CA125 that reacted predominantly with malignant ovarian tissue [9]. Almost 80% of advanced OC cases (stage III or IV) have elevated serum CA125 levels at diagnosis [10]. Research following these findings demonstrated that serum CA125 levels correlate with both disease stage and response to chemotherapy, suggesting that CA125 may be helpful as a marker of disease progression and a biomarker of prognosis. The potential use of CA125 as a tool in early detection was extrapolated from these studies, and case reports noted its increase in asymptomatic women before being diagnosed with ovarian cancer [11].

The biology behind the apparent association of CA125 with OC risk is unclear; however, it has been suggested that CA125 may play a role in peritoneal cavity metastasis, but these findings have not been replicated clinically [12,13]. Its limitations are its low sensitivity in early stages in only 50% of the cases and its low specificity, as it is increased in other pathologies. In fact, CA125 may be markedly elevated in patients with a variety of benign conditions, such as endometriosis or non-ovarian malignancies, and in approximately 20% of OC cases, it is not expressed at all or only in small amounts [14]. Serial determinations of CA125, using algorithms that incorporate age and rate of increase of the marker, improve its PPV (positive predictive value), but probably not enough to be incorporated into daily clinical practice [1,4].

### 3.2. Tumour Marker HE4

In 1999, the WFDC2 (WAP four-disulphide core domain protein 2), which encodes the HE4 protein, was detected as a potential diagnostic marker for CO [15]. Compared to CA125, HE4 has a similar sensitivity for detecting late-stage OC, but a higher specificity in differentiating between malignant and benign tumours [16]. As with CA125, elevated serum HE4 levels are not unique to women with ovarian tumours and are found in individuals with tumours of gynaecological and pulmonary origin [17]. Nevertheless, dramatic increases in serum HE4 concentration observed in women with OC (and in the serous and endometrioid subtypes in particular) support its usefulness as a biomarker with high specificity for early detection of CO [8,18]. 

### 3.3. HE4 + CA125 Association

There are three meta-analyses or systematic reviews showing the use of HE4 or CA125, always with different thresholds for HE4 [19,20,21]. However, the combined use of these markers is only sometimes studied [21]. These authors reported a specificity of 65.7% using the ECLIA (electro-chemo-luminescence) immunological method with a cut-off value for HE4 of 140 pmol/L. In a different study using another technique to assess serum HE4, the specificity of the CA125 and HE4 association was much better (80%) [22]. However, the area under the ROC curve when combining the two markers was high, ranging from 0.96 (95% CI 0.93–1.00) to 0.91 (95% CI 86.7–96.0) [23]. Furthermore, while HE4 varies in women smokers and users of combined contraceptives, simultaneous assessment of CA125, which is not affected by these variables, should allow better interpretation of abnormal HE4 levels [24].

In conclusion, it seems more useful to measure both markers in cases of suspected ovarian tumours; an increased value of both markers suggests CO, as suggested by a recent study [25]. Thresholds of 70 and 140 pmol/L depending on menopausal status seem preferable for HE4 and 35 IU/mL for CA125. Table 1 shows a summary of the diagnostic performance of CA125, HE4, the combination of CA125 + HE4, RMI (Risk Malignancy Index) and ROMA (Risk Ovarian Malignancy Algorithm) [26], expressing the characteristics of sensitivity, specificity, positive predictive value and negative predictive value of each biomarker, of the combinations between them and of the strategies of the different algorithms that are expressed [24,26].

### 3.4. Multimodality Screening 

Due to the marked heterogeneity of OC cases, no single tumour biomarker is likely to be enough to aid in the early detection of all histological subtypes. Research shows that different carcinoma subtypes express different sets of proteins [30]. There are also questions about the timing and type of patient samples collected in clinical trials, although this screening trial suggests that serial biomarker measurements have better predictive power than single-point sampling.

Another outstanding challenge is determining which marker or combination of markers meets the sensitivity and specificity requirements for early detection of a rare and heterogeneous disease. The difficulties in performing these validation studies are intensified by OC’s low incidence, especially when separating the different subtypes. While other cancers also have multiple subtypes, OC differs in that the various subtypes are likely to develop from different tissues of origin. Several important trials have been conducted to determine whether screening for OC in the high-risk or general population reduces mortality from the disease [26].

### 3.5. Trial Description

The randomised Prostate, Lung, Colorectal and Ovarian Cancer Screening (PLCO) trial included 78,216 women in the USA; it concluded that screening with transvaginal ultrasound (TVUS) and CA125 does not decrease OC mortality. There was also no difference in diagnosis at earlier stages between the screening group and the control group [31]. However, an increase in invasive medical procedures and associated harms were observed. A limitation in earlier studies and a limitation of the one carried out here was using a single fixed cut-off for serum CA125 levels. Follow-up studies of the PLCO study results suggest that almost 20% of OC could have been detected at an earlier stage if a serial trajectory of CA125 level had been considered.

In a British study that evaluated screening for OC (UKCTOCS), 202,638 women were randomised to multimodal screening with TVU and CA125, TVU alone or no screening. After a median follow-up of eleven years, the reduction in OC mortality was not significant in the primary analysis. However, a significant reduction in mortality was observed with multimodality screening when prevalent cases were excluded. The authors concluded that further follow-up is needed to determine the efficacy and cost-effectiveness of OC screening [32] (it is estimated that 641 women should be screened annually for 14 years to prevent OC death). In this study, sequential CA125 measurements taken at annual screening visits are used to assess the trajectory of serum CA125 overtime after an age-adjusted baseline measurement using the Risk of Ovarian Cancer Algorithm (ROCA). It assigns a risk level based on CA125 levels and changes over time combined with age and known OC risk factors for each patient. If the algorithm indicates increased risk, transvaginal ultrasound is indicated as a secondary test [32].

In 2016, the US Food and Drug Administration (FDA) issued a recommendation against the use of commercially available tests for OC screening. FDA stated that there are no FDA-approved OC screening tests, and there is no published clinical data to demonstrate that currently available tests are accurate and reliable in asymptomatic women. FDA stated that the ROCA algorithm was marketed in the US without data to support its claims for OC detection and improved cancer survival [33]. Following this FDA statement, the company marketing the ROCA test discontinued its commercial availability in the US [34].

Additionally, data from a trial in Japan from the Shizuoka cohort of prospective, randomised, controlled trials are available. The study included 83,000 menopausal women who were randomised to annual screening with VTE and CA125 versus no screening. The study closed in December 2002 with a median follow-up period of 9.2 years. A higher detection of early stages was observed in the screening group without reaching statistical significance. No survival data is available [35]. Table 2 shows a summary of results from prospective OC screening trials. 

### 3.6. Early Detection in the High-Risk Population 

There are no randomised controlled trials for this type of high-risk patients. The results of follow-up programmes are not encouraging. In the UKFOCSS (United Kingdom Familial Ovarian Cancer Screening Study), 3563 women with previous family OC syndrome were followed annually with VTE and CA125. The study was not designed to examine whether screening decreased mortality. However, detection of early stage cancer in women who adhered to screening has reduced the screening interval to four months in phase II of this trial. In addition, the UKCTOCS algorithm will also be incorporated in this next phase for follow-up of abnormal CA125 results. Although prophylactic bilateral salpingo-oophorectomy (PBSO) remains the only method of reducing OC mortality in the high-risk population, screening may reduce risk until surgery [36].

### 3.7. Tests Based on Prognostic and Predictive Biomarkers in Patients with Symptoms

These tests may be based on a single biomarker or may assess a platform of multiple biomarkers (e.g., omics-based tests, which often combine multiple biomarker values from omics assays according to some algorithm or mathematical model). Prognostic and predictive tests should be carefully evaluated to determine whether their use leads to better treatment decisions and benefits for patients [37].

### 3.8. New Screening Tools

There is no information on new ongoing randomised trials of OC screening using new screening tools. There are currently no new screening tools that show performance levels beyond what has been observed for the screening tools evaluated in previous trials. Efforts are underway to improve the ROCA algorithm by adding more protein markers along with CA125 using data from the UKCTOCS clinical trial [32,36]. Given the absence of a single marker or screening device effective for diagnosis OC, it is likely that research will increasingly aim to identify new markers and marker combinations to generate predictive models. A possible alternative application of biomarker panels, once they have undergone further validation, would be to use them as a screening tool for clinicians to assess out of a single blood sample whether a symptomatic patient is at low or high risk of OC. In this context, it may be a helpful tool on its own or in combination with current protocols used for the differential diagnosis of an adnexal mass in symptomatic patients.

There is a definite need to define and identify high-risk populations for the development of OC and screen these patients with transvaginal ultrasound and serum CA125 determination every six months [38].

### 3.9. Risk of Malignancy Index (RMI)

RMI combines age, ultrasound examination score, menopausal status, a clinical history score and serum CA125 level to better discriminate between patients with benign (*n* = 101) and malignant (*n* = 42) pelvic masses. Each criterion used alone provided statistically significant discrimination. The most useful individual criteria were a serum CA125 level of 30 U/mL (sensitivity 81%, specificity 75%) and an ultrasound score of 2 (sensitivity 71%, specificity 83%). Three criteria could be combined into an RMI risk that is simply calculated using the product of serum CA125 level (U/mL), ultrasound result (expressed as a score of 0, 1 or 3) and menopausal status 1, if premenopausal (Pre-M) and 3, if postmenopausal (Post-M). This index was statistically as effective as previous methods to discriminate between cancer and benign lesions. Using an RMI cut-off level of 200, the sensitivity was 85%, and the specificity was 97%. Patients with an RMI score above 200 had, on average, 42 times the baseline risk of cancer and those with a lower value 0.15 times the baseline risk [39]. The RMI score is calculated according to the criteria of Tingulstad et al. [40]. RMI = U × M × serum CA125, where U = Ultrasound of 1 for imaging score 0–1 and U = 3 for an imaging score of 2–5; M = Menopausal status, M = 1 if Pre-M and M = 3 if Post-M. The cut-off point is 200.

### 3.10. ROMA Algorithm

The Risk of Ovarian Malignancy Algorithm (ROMA) was proposed by Moore et al. [41]. The aim of this trial was to validate a predictive model to assess the risk for ovarian epithelial cancer (OEC) in women with a pelvic mass. Women diagnosed with a pelvic mass and scheduled for surgery were enrolled in a prospective multicentre study. Preoperative serum HE4 and CA125 levels were measured. Separate logistic regression algorithms for premenopausal and postmenopausal women were used to classify patients into low- and high-risk groups for CEO. The ROMA score was calculated according to: Pre-Menopausal predictive index (PI) = −12.0 + 2.38 × LN (HE4) + 0.0626 × LN (CA125); Post-Menopausal PI = −8.09 + 1.04 × LN (HE4) + 0.732 × LN (CA125). The predicted probability was calculated as ROMA% = exp. (PI)/(1 + exp. (PI)). Cut-off levels were used for Pre-M ≥ 11.4% and Post-M ≥ 29.9% [41].

The postmenopausal group had 150 benign cases, of which 112 were classified as low risk giving a specificity of 75.0% (95% CI 66.9–81.4), and 111 OEC tumours and 6 LMP (low malignant potential), of which 108 were classified as high risk giving a sensitivity of 92.3% (95% CI 85.9–96.4). The premenopausal group had 202 benign cases, of which 151 were classified as low risk, giving a specificity of 74.8% (95% CI 68.2–80.6), and 18 OEC tumours and 16 LMP, of which 26 were classified as high risk, with a sensitivity of 76.5% (95% CI 58.8–89.3).

Combining markers improves the performance of CA125 alone without using predefined cut-offs for serum tumour markers. Furthermore, for the 20% of OECs that express little or no CA125, a single marker is not sufficient. In particular, HE4 levels are elevated in more than 50% of tumours that do not express CA125 [42]. Therefore, the addition of HE4 to CA125 allows detecting malignancies in patients with tumours that do not express CA125 and will be missed by algorithms using CA125 alone. Similarly, a combination of HE4 and CA125 or HE4 alone has been shown to have a higher sensitivity in patients with the early stage disease compared to CA125.

### 3.11. OVA 1 Test

The OVA1 test, approved by the FDA (Foods and Drugs Administration), is used as a guide for gynaecological oncologists’ referral. It is a qualitative serum test that combines the results of five immunoassays (CA125 and apolipoprotein A1, transthyretin, transferrin and b2-microglobulin) into a single numerical score for women with an ovarian adnexal mass for whom surgery is planned. It helps assess the likelihood of malignancy further when independent clinical and radiological assessment does not indicate malignancy [43]. It is a first-generation multivariate index assay (MIA) that could minimise the uncertainty of a pre-surgical study of adnexal masses. It is essentially an FDA-approved blood test that assesses malignancy risk in adnexal masses planned for surgery [44]. The objective was to validate the efficacy of a multivariate assay in identifying ovarian malignancy compared to clinical assessment and CA125-II among women undergoing surgery for an adnexal mass after referral by non-oncological gynaecologists.

For all ovarian malignancies, the multivariate assay’s sensitivity was 95.7% (95% CI 89.3–98.3) when combined with clinical impression. The multivariate assay correctly predicted ovarian malignancy in 91.4% (95% CI 77.6–97.0) of early stage disease cases, compared with 65.7% (95% CI 49.2–79.2) for CA125-II. The multivariate assay correctly identified 83.3% of malignancies missed by clinical impression and 70.8% of cases missed by CA125-II. It was superior in predicting the absence of ovarian malignancy, with a negative predictive value of 98.1% (95% CI 95.2–99.2). Both clinical impression and CA125-II were more accurate in identifying benign disease [44].

More recently, in 2016, the FDA approved the Ova1 (OVERA) test with CA 125-II, HE4, apolipoprotein A-1, FSH and transferrin (sensitivity 91% and specificity 69%) as a reference or screening test in patients presenting ovarian masses [45].

### 3.12. Improving the Performance of Additional Biomarkers

Active areas of OC research focus their activity on identifying new markers to improve the ability to detect cancers at earlier stages and, more importantly, for screening, if possible. High processing technology has produced multiple biomarkers associated with OC. The Early Detection Research Network (EDRN), an initiative of the National Cancer Institute (NCI) [46], brings together dozens of institutions to help accelerate the translation of biomarker information into clinical applications and to evaluate new ways to test for cancer at its earliest stages and to determine cancer risk. At present, in EDRN, there are already a total of 204 biomarkers under study.

The question is, if additional biomarkers can improve performance, what is their ability to predict OEC in women with a pelvic mass? This other study by Moore et al. [47] examines the performance of eight serum biomarkers that have been well documented to be active biomarkers of OC, including six biomarkers currently used in two previously described algorithms, as well as YKL40 (also called Chitinase 3-like 1) and lysofosphagic acid (LPA). The study aimed to determine the optimal combination of biomarkers to predict malignancy in women presenting with a pelvic mass. Women who met the inclusion criteria underwent serum measurement of biomarkers CA125, HE4, YKL-40, transthyretin, Apolipoprotein A1 (ApoA1), Beta 2 microglobulin, transferrin and APL before undergoing surgery. The primary endpoint of the study was to determine the optimal combination of biomarkers to predict cancer. The comparison of ROMA versus OVA1 was not directly performed in this analysis, as the OVA1 algorithm has never been published and, as such, has not been publicly available for independent validation. ROMA, the ovarian malignancy algorithm, has been published and is available for independent analysis and validation. To summarise the main results of this study^44^, a diagnostic performance of the biomarker combinations for differentiation of benign tumours (*n* = 103) vs. OEC (*n* = 61) compared to ROMA is shown in Table 3 and Table 4, the results of predictive statistics for ROMA and the combination of CA125, HE4, YKL-40, transthyretin, ApoA1, Beta 2-microglobulin, transferrin, LPA and menopausal status (8-marker assay) for OEC versus benign disease at a specificity level of ~75% are shown.

This study found that the dual marker combination of HE4 and CA125 (ROMA) to predict malignancy performed as well as any other biomarker combination. Increasing the number of biomarkers used in a clinical trial can significantly increase the test’s cost without creating a clinically meaningful increase in sensitivity and/or specificity. Thus, the addition of an increasing number of biomarkers in an algorithm to generally differentiate benign from malignant tumours only serves to minimally increase the sensitivity of the test while negatively affecting specificity. The present study found no value in adding additional biomarkers to CA125 and HE4 as used in the ROMA test [47].

## 4. Discussion 

Actually, there is no clinical, radiological or analytical test that meets the necessary requirements to be considered for early detection of OC in the general population (moderate evidence, with a strongly opposed recommendation).

It is well known that the high mortality rate of OC is mainly due to the absence of specific symptoms, which means that it is mainly diagnosed in advanced stages and lacks effective early detection methods. It is a disease that predominantly affects menopausal women; more than 80% of cases are diagnosed in women over 50 years of age [48]. 

Although most cancers affecting the ovaries are called ovarian cancer, many cancers may not originate in the ovaries. Even those that originate in the ovaries may arise from cell types that are not considered intrinsic to normal ovaries, such as endometrial-type epithelium or fallopian tube epithelium [49,50]. An incomplete understanding of the origins of each type of OC may hinder the development of effective methods of prevention, early detection, and treatment. Indeed, early detection of OCs may require looking at sites other than the ovaries themselves because a growing evidence base suggests that many ovarian carcinomas arise from extra-ovarian sites and tissues and spread to the ovaries secondarily.

ROMA is arguably helpful in distinguishing epithelial ovarian cancer from benign pelvic mass, but HE4 is no better than CA125 for either OEC or OC prediction. ROMA is a promising predictor of ovarian epithelial cancer to replace CA125, but its use requires further exploration [27,28,47]. Other authors have confirmed that HE4 and ROMA had very high specificity but lower sensitivity than CA125 and RMI, at least in premenopausal women; however, they showed comparable sensitivity in postmenopausal women and proved valuable in distinguishing benign ovarian tumours or endometriosis from OC [29].

Secondary prevention is prevention in the pathogenic period when the disease is anatomically detectable. Two periods can be identified: pre-symptomatic and symptomatic. Interventions performed in this period do not decrease the incidence of the disease. However, initiating treatment in the early stages leads to a better course of the disease and may reduce mortality. Secondary prevention measures are aimed at early diagnosis of incipient disease without clinical manifestations. It means the search for disease in apparently healthy subjects to detect it as early as possible. Mainly represented by screening or disease detection actions, also called early diagnosis, monitoring or screening.

In the case of OC, these actions aim to make early diagnoses that increase survival, avoid or delay the appearance of sequelae, artificially changing their evolution to cure, mitigating sequelae and trying to avoid death. The detection of healthy people as sick (false positives) is the main disadvantage of screening methods, as they expose healthy people to complementary tests that confirm or rule out the diagnosis. However, we cannot ignore the discomfort and/or risks of the test performed, overdiagnosis or the diagnosis of unsuspected lesions [8,13,26,32,33].

Cancer prevalence, which should be the target of screening, is the number of patients with the disease in a population independent when diagnosed, being a measure that depends on incidence and survival. It is a useful indicator for planning care resources according to the burden of disease in the population. Prevalence is determined by survival, i.e., prevalence is higher in tumours with more prolonged survival, while tumours with shorter survival may have a lower prevalence, even though they are diagnosed more frequently [50].

In the case of OC and due to its low prevalence, a practical screening test for this neoplasm would require a sensitivity more significant than 75% and a specificity greater than 99.6% to achieve a positive predictive value (PPV) of 10%, among other requirements [51,52]. It is a standard objective not to perform more than 10 exploratory diagnostic operations for each real case of OC [52]. The concern is the detection of false positives leading to unnecessary surgical techniques. Setting these minimum targets is essential to avoid an unacceptable level of unnecessary and potentially harmful surgical or chemotherapeutic interventions [53].

Detection of disease in the absence of symptoms is a key tool in early detection. In general, the effectiveness of a screening test is evaluated in terms of its ability to identify those individuals who have the disease in question and rule out those individuals who most certainly do not [50,53].

The markers that we manage actually have many limitations, but they are currently validated. Research is currently working on proteomic or metabolic techniques that try to discover alternative biomarkers obtained in a minimally invasive way in urine, serum or plasma samples, trying to detect molecules that can serve as an early diagnosis of ovarian cancer or for the early detection of relapses.[54]

## 5. Conclusions

Currently, there is no clinical, radiological, ultrasound or analytical test that meets the requirements to be considered early detection for OC in the general population. Randomised trials yield inconsistent results, even though the screening and follow-up methods were different, and the extent of any possible benefit seems small. There is no reliable screening method to detect OC at an early stage, and, as a result, no professional organisation recommends screening in the general population. The combination of HE4 and CA125 is sensitive, but not specific for differentiating a benign from a malignant adnexal mass and predicting OC. Additional markers to HE4 and CA125 do not significantly improve the sensitivity of OC detection. HE4 and CA125 are complementary biomarkers and work best when used in combination. ROMA appears to be a promising predictor of epithelial ovarian cancer to replace CA125, but its clinical use requires further investigation.

## Figures and Tables

**Table 1:** Diagnostic performance of CA125, HE4, the combination of CA125 + HE4, RMI and ROMA (modif. from reference [24]).

Reference. Author and Year	The Systemic Revision or Metanalysis	CA125					HE4					CA125 + HE4				
		Se (%) (95% IC)	Sp (%) (95% IC)	PPV (%)	NPV (%)	AUC (95% IC)	Se (%) (95% IC)	Sp (%) (95% IC)	PPV (%)	NPV (%)	AUC (95% IC)	Se (%) (95% IC)	Sp (%) (95% IC)	PPV (%)	NPV (%)	AUC (95% IC)
[19] Ferraro S. et al., 2013	X	79 (77–82)	78 (76–80)				79 (76–81)	93 (92–94)				82 (78–86)	76 (72–80)			
[20] Wang J et al. 2014	X	79 (74–84)	82 (77–87)			0.87 (0.84–0.90)	76 (72–80)	94 (90–96)			0.89 (0.86–0.92)					
[21] Zhen et al. 2014	X	74 (72–76)	83 (81–84)			0.85	74 (72–76)	90 (89–91)			0.89					
[27] Meys et al. 2016	X															
[28] Li et al. 2012	X	77 (58–89)	84 (76–90)			0.88 (0.85–0.91)	79 (74–84)	93 (87–96)			0.82 (0.78–0.85)					
		RMI					ROMA									
[20] Wang J et al. 2014	X						85 (81–89)	82 (77–87)			0.91 (0.88–0.93)					
[27] Meys et al. 2016	X	75 (72–79)	92 (88–94)													
Al Musalhi et al. [29] 2016	X	77	82	56	93	0.85	75	88	65	92	0.84					
[28] Li et al. 2012	X						89 (84–93)	83 (77–88)			0.93 (0.90–0.95)					

CA125: carbohydrate antigen 125, HE4: human epididymal protein 4, Se: sensitivity, Sp: specificity, PPV: positive predictive value, NPV: negative predictive value, AUC: area under the curve, and CI: confidence interval. ROMA: risk of ovarian malignancy algorithm. RMI: Risk of Malignancy Index for Ovarian Cancer.

**Table 2:** Summary of the results of ovarian cancer detection trials.

Name of the Study	Study Design	Screening Cohort Size	Screening Strategy	Interpretation CA125	Sensitivity (%)	Mortality/Subrogated Results	Author, Year. (Reference)
PLCO	RCT, on general population	30,630	CA125 + TVU	Fixed cut, 35 U/mL	IOC/FT: 69.5 IOC/FT: 68.2	No mortality benefit	Buys et al. 2011 [31]
UKCTO	RCT, on	101,247	1.	ROCA	OC/FT:	Relative	Jacobs
CS	general population,		CA125 following	longitudinal sampling	MMS (89.4)/	mortality reduction of	et al. 2015 [32]
	two arms		for MMS		TVU (84.9)	MMS (14%)	
					IOC/FT:	and groups of	
					MMS	USS (11%)	
			2. Only		(84.9)/	about no	
			TVU		TVU (75.0)	action, but	
					IOC/FT:	the reductions	
					MMS	were not	
					(88.6)/	significant in	
					TVU (65.8)	the primary	
						analysis	
Japanes e cohort Shizuoka	RCT, low risk post-menop ausal	41,688	Physical examinat ion, CA125 and TVU	Fixed cut, 35 U/mL	OC/FT: 77.1	Change of stage; Stage I OC in screening (63%) versus	Kobayashi et al. 2008 [35]
						Control (38%)	

%: percentage. PLCO: Prostate, Lung, Colorectal and Ovarian. RCT: randomised clinical trial. TVU: transvaginal ultrasound. IOC/FT: invasive ovarian or fallopian tube cancer. OC/FT = ovarian or fallopian tube cancer. UKCTOCS: United Kingdom Collaborative Trial of Ovarian Cancer Screening. UK collaborative trial of OC screening. MMS = multimodality screening. ROCA: Risk of Ovarian Cancer Algorithm. Risk of Ovarian Cancer Algorithm. USS = ultrasound screening.

**Table 3:** Diagnostic performance of biomarker combinations for differencing benign tumours (*n* = 103) vs. OEC (*n* = 61) compared to ROMA (modified from reference [47]).

Evaluated Marker or Combination of Markers	ROC-AUC (95% CI)	*p* Value vs. ROMA
CA125	86.6% (80.6–92.6%)	0.039
HE4	90.8% (85.7–95.8%)	0.671
ROMA (CA-125 + HE4 + menopausal)	91.2% (86.0–96.4%)	–––
CA125 + Transthyretin + ApoA1 + Beta2 Microglobulin + Transferrin + menopausal	89.7% (84.4–95.0%)	0.502
CA125 + HE4 + ApoA1 + Transferrin + menopausal	93.2% (88.7–97.6%)	0.153
CA125 + HE4 + YKL-40 + Transthyretin + ApoA1 + Beta2 Microglobulin + Transferrin + LPA + menopausal	94.6% (90.1–99.2%)	0.078

OEC: ovarian epithelial cancer. ROC-AUC: area under the ROC curve. 95% CI: 95% confidence interval. ROMA: Risk of Ovarian Malignancy Algorithm (from Risk of Ovarian Malignancy Algorithm). ApoA1: Apolipoprotein A1. YKL-40: Glicoprotein (chinitase family). LPA: Lysophosphatidic Acid

**Table 4:** Predictive statistics for ROMA and the combination of CA125, HE4, YKL-40, transthyretin, ApoA1, Beta 2 microglobulin, transferrin, LPA, and menopausal status (8-marker assay) for OEC versus benign disease at a specificity level of ~ 75% (modify from [44]).

Analysed Value	ROMA (95% CI)	8 Markers Trial (95% CI)
Sensitivity	90.0% (79.5–96.2%)	94.0% (83.5–98.7%)
Specificity	76.7% (67.3–84.5%)	76.3% (66.4–84.5%)
PPV	69.2% (57.8–79.2%)	68.1% (55.8–78.8%)
NPV	92.9% (85.3–97.4%)	95.9% (88.6–99.2%)
Accuracy	81.6% (74.8–87.2%)	82.5% (75.3–88.4%)

OEC: ovarian epithelial cancer. ROMA: Risk of Ovarian Malignancy Algorithm (from Risk of Ovarian Malignancy Algorithm). CI 95% 95% confidence interval PPV: Positive predicted value. NPV: Negative predicted value.

## Data Availability

The data that support the findings of this study are available from the corresponding author upon reasonable request.
